# Extra-axial primary meningeal pleomorphic xanthoastrocytoma: a case report

**DOI:** 10.11604/pamj.2024.47.220.39924

**Published:** 2024-04-30

**Authors:** Emna Mili, Kais Maamri, Mohamed Amine Hadj Taieb, Mohamed Boukhit, Zohra Souei, Amine Trifa, Mohamed Maher Hadhri, Mehdi Darmoul

**Affiliations:** 1 Anesthesia Department, Fatouma Bourguiba Hospital, Monastir, Tunisia,; 2 Neurosurgery Department, Fatouma Bourguiba Hospital, Monastir, Tunisia

**Keywords:** Glioma, extra-axial, meningioma-like, pleomorphic xanthoastrocytoma, case report

## Abstract

Pleomorphic xanthoastrocytoma (PXA) is a rare low-grade glial neoplasm of the central nervous system accounting for less than 1% of all astrocytomas. Similar to other gliomas, it can rarely arise from glial nests in the meninges, manifesting as an extra-axial mass mimicking a meningioma. Extra axial PXA is an extremely rare entity. Therefore, there are no standardized guidelines. In this article, we report the fourth case, so far, of a solitary primary extra-axial PXA mimicking a meningioma in a 23-year-old woman who presented with temporal seizures and features of raised intracranial pressure. Through this case, we tried to discuss all treatment options.

## Introduction

Pleomorphic xanthoastrocytoma (PXA) is an uncommon astrocytic tumor accounting for less than 1% of all astrocytomas [1]. Kepes was the first to describe it as a distinct entity in 1979 [2]. In 1993, it was formally incorporated into the World Health Organization (WHO) classification system of central nervous system tumors as a WHO grade II tumor. In 2007, it was considered a grade II benign glioma, with the possibility of signs of anaplasia. Finally, in the 2016 edition, PXAs with anaplastic features were upgraded to grade III named “anaplastic PXA”.

Histologically, PXA is characterized by pleomorphic and lipidized cells, multinucleated giant cells, and eosinophilic granular bodies in an inflammatory background. Similar to other gliomas, it rarely presents as an extra-axial mass mimicking a meningioma.

We report a case of a solitary extra-axial PXA in a 23-year-old woman.

## Patient and observation

**Patient information:** a 23-year-old female patient, with no medical history, non-smoker, non-alcoholic.

**Clinical findings:** the patient presented to our emergency with repeated episodes of a rising sick feeling in her stomach, similar to being on a roller coaster followed by a short period of confusion and difficulty speaking. On admission, a physical examination showed no abnormalities. The patient had no motor or sensory deficits. Dilated fundus examination was normal.

**Timeline of the current episode:** April 2021: gross total resection (GTR), histology and immunohistochemical study were conducted.

**Figure 1:**
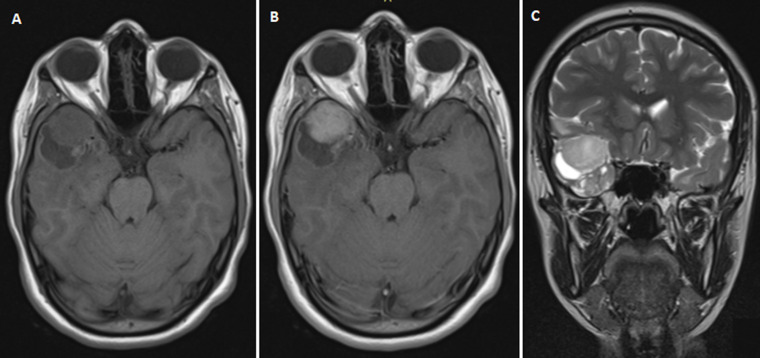
preoperative magnetic resonance imaging of the brain showing an extra-axial lesion in the right temporal fossa with two components solid and cystic (A) isointense on T1 weighted-images, (B) heterogeneously enhanced in T1 weighted-images, and (C) isointense on T2 weighted-images

**Diagnostic assessment:** we suspected the diagnosis of temporal seizures. Therefore, a brain magnetic resonance imaging (MRI) was performed revealing an extra-axial dura-based lesion in the right temporal fossa. The tumor had two components solid and cystic. The solid mass measuring 22*28*20 mm was isointense on T1-weighted images (T1w), isointense on T2-weighted images (T2w), and heterogeneously enhanced (Figure 1).

**Therapeutic interventions:** after the preoperative evaluation, the patient underwent surgery. A right temporal craniotomy was performed. Intraoperatively, the tumor was extra-axial, grayish-white, and highly vascular. We were able to achieve a gross total resection since the tumor was well-demarcated from the surrounding brain.

**Figure 2:**
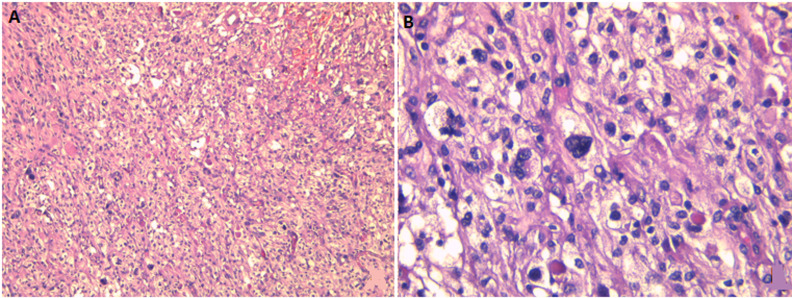
histopathological examination: (A) mixed population of spindled and epithelioid cells (Hematoxylin Eosin X100); (B) pleomorphic and xanthomatous cells with intracytoplasmic eosinophilic granular bodies (Hematoxylin Eosin X400)

**Figure 3:**
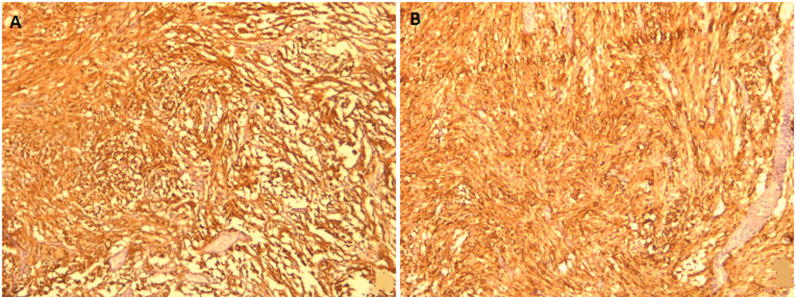
immunohistochemical examination: (A) positive immunostaining for S100 protein (X100); (B) diffuse positive immunostaining for glial fibrillary acid protein (X100)

**Diagnosis:** on histopathological examination (Figure 2), hematoxylin and eosin-stained sections showed a tumor composed of spindle and epithelioid cells arranged in sheets and fascicles admixed multinucleated giant cells. The neoplastic cells had multivacuolated foamy cytoplasm and hyperchromatic nuclei with anisonucleosis. There were no mitoses or necrosis. On the immunohistochemical examination (Figure 3), the neoplastic cells were positive for glial fibrillary acidic protein (GFAP), PS100, vimentin, and reticulin. In conclusion, the final diagnosis was a temporal extra-axial PXA.

**Follow-up and outcome of interventions:** the postoperative course was uneventful and the patient was seizure-free. She was discharged from the hospital three days later with no adjuvant therapy.

**Figure 4:**
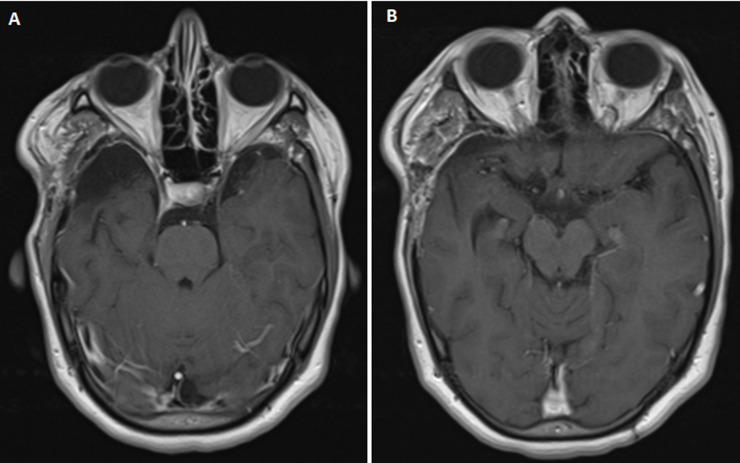
(A,B) two-year follow-up magnetic resonance imaging of the brain: no recurrence

**Patient perspective:** during a follow-up period of 2 years, the patient remained seizure-free. A postoperative MRI of the brain performed 18 months after surgery, confirmed the absence of tumor recurrence (Figure 4).

**Informed consent:** written informed consent was obtained from the patient to publish this report by the journal's patient consent policy.

## Discussion

Intracranial gliomas arising primarily in the leptomeninges are rare. These primary meningeal gliomas (PMG) may occur either as a solitary mass or as diffuse leptomeningeal gliomatosis. We reviewed the relevant literature and identified only 31 cases of a solitary meningeal glial mass reported in English literature [3]. Most PMG were astrocytomas (53.3%) followed by glioblastomas (30%) [3,4]. To our knowledge, we are presenting the fourth case of an extra-axial PXA (Table 1). In our case, the preoperative imaging studies and the intraoperative findings of an extra-axial mass arising from the dura with the lack of adhesion to adjacent brain tissue in addition to the relative ease of surgical removal of the tumor made the diagnosis of meningioma very likely. However, the histopathological examination confirmed the diagnosis of PXA.

**Table 1:** information summary of the reported cases of primary meningeal pleomorphic xanthoastrocytoma (PXA) in English literature

Author, year	Age (years)/sex	Localization	Surgery	Pathology	Outcome
Usubalieva, 2015	56/F	Left tentorial	GTR	Anaplastic PXA	Alive 37 months after surgery
	35/F	Left tentorial	STR	Anaplastic PXA	Died 26 months after the presentation
Dadhich, 2019	9/F	Right tentorial	STR	PXA	Alive 6 months after presentation
Present case, 2022	23/F	Right temporal fossa	GTR	PXA	Alive 2 years after surgery with no recurrence on MRI

F: female; GTR: gross total resection; STR: subtotal resection; PXA: pleomorphic xanthoastrocytoma; MRI: magnetic resonance imaging

Typically, PXA consists of solid and cystic components on imaging. In contrast-enhanced computed tomography imaging, the cystic component is hypodense. The solid component typically appears hypo to isodense, but can occasionally be hyperdense, with areas of calcification seen in the solid component [1]. However, it can be presented as a superficial mass with leptomeningeal contact [3,5]. Therefore, these tumors should be considered as a differential diagnosis of intracranial meningiomas.

Cooper and Kernohan considered in 1951 that heterotopic glial nests are the foci of origin of solitary PMG [6]. On its part, Kepes *et al*. originally suggested that PXAs involving the leptomeninges may be derived from subpial astrocytes [2]. Recently, according to the cancer stem cell theory, pluripotent neural progenitor cells which derive from radial glia or neural crest cells are implicated in the development of extra-axial PXAs [4]. All the reported cases of solitary primary meningeal PXA satisfied the diagnostic criteria of PMG formulated by Cooper and Kernohan [6] namely the absence of intra-axial tumor and parenchymal invasion, and the presence of a sheath encapsulating the tumor. In all the cases, the tumor developed in the supratentorial space: two from the tentorium, one from the frontal lobe, and in the present cases from the temporal lobe. On the other hand, in the two cases reported by Usubalieva *et al*., the tumor showed anaplastic features while in our case and Dadhich´s *et al*. no malignant criteria were observed [3,4].

The absence of recurrence in our patient after two years of follow-up reinforces the theory elaborated by Kepes *et al*. suggesting that the most determinant factors of a favorable prognosis are the youth age and the ease of surgical removal [2].

Due to the lack of reported data, there are no formal guidelines published on the management of PMGs. Treatment options include surgical resection followed by adjuvant therapy including radiotherapy with or without systemic therapy. In managing parenchymal PXAs, multiple studies concluded that the extent of resection is a reliable prognostic factor for overall survival (OS) and progression-free survival (PFS). Therefore, gross total resection (GTR) should be the goal of the surgeon [1,7]. Adjuvant therapy has been generally considered minimally effective or ineffective for the treatment of PXAs. A meta-analysis of 167 patients with grade II PXA did not demonstrate an association between adjuvant therapy with improved oncologic outcomes [7]. For primary meningeal PXAs, all reported cases were treated with surgery, 2 cases with subtotal resection, and 1 case with gross total resection.

The overall prognosis of PXA is relatively favorable. The OS of PXA is favorable with 5-year survival rates of >75%. However, monitoring for recurrence and malignant transformation is warranted since the recurrence rate of PXA following surgical resection is about 30% within 5 years and 40% within 10 years [8], and 10-20% undergo an anaplastic transformation [3]. In his meta-analysis of 167 patients, Mallick *et al*. found that the estimated median OS was 209 months, and the estimated median PFS was 48 months [7].

## Conclusion

Solitary extra-axial dura-based PXA is an extremely rare entity. Therefore, there are no standardized guidelines for the management of solitary extra-axial primary meningeal PXA. However, as it is for parenchymal PXA, it is plausible that the extent of surgical resection is strongly predictive of recurrence-free survival.
